# Association Between Oxygen Partial Pressure Trajectories and Short-Term Outcomes in Patients With Hemorrhagic Brain Injury

**DOI:** 10.3389/fmed.2021.681200

**Published:** 2021-09-09

**Authors:** Guolong Cai, Weizhe Ru, Qianghong Xu, Jiong Wu, Shijin Gong, Jing Yan, Yanfei Shen

**Affiliations:** ^1^Department of Intensive Care, Zhejiang Hospital, Hangzhou, China; ^2^Department of Oncology, Cixi People's Hospital, Cixi, China; ^3^Department of Neurology, Zhejiang Hospital, Hangzhou, China

**Keywords:** Glasgow Coma Scale, hyperoxia, hospital mortality, intracranial hemorrhage, oxygen partial pressure

## Abstract

**Objectives:** Arterial hyperoxia is reportedly a risk factor for poor outcomes in patients with hemorrhagic brain injury (HBI). However, most previous studies have only evaluated the effects of hyperoxia using static oxygen partial pressure (PaO_2_) values. This study aimed to investigate the association between overall dynamic oxygenation status and HBI outcomes, using longitudinal PaO_2_ data.

**Methods:** Data were extracted from the Medical Information Mart for Intensive Care III database. Longitudinal PaO_2_ data obtained within 72 h of admission to an intensive care unit were analyzed, using a group-based trajectory approach. In-hospital mortality was used as the primary outcomes. Multivariable logistic models were used to explore the association between PaO_2_ trajectory and outcomes.

**Results:** Data of 2,028 patients with HBI were analyzed. Three PaO_2_ trajectory types were identified: Traj-1 (mild hyperoxia), Traj-2 (transient severe hyperoxia), and Traj-3 (persistent severe hyperoxia). The initial and maximum PaO_2_ of patients with Traj-2 and Traj-3 were similar and significantly higher than those of patients with Traj-1. However, PaO_2_ in patients with Traj-2 decreased more rapidly than in patients with Traj-3. The crude in-hospital mortality was the lowest for patients with Traj-1 and highest for patients with Traj-3 (365/1,303, 209/640, and 43/85 for Traj-1, Traj-2, and Traj-3, respectively; *p* < 0.001), and the mean Glasgow Coma Scale score at discharge (GCS_dis_) was highest for patients with Traj-1 and lowest in patients with Traj-3 (13 [7–15], 11 [6–15], and 7 [3–14] for Traj-1, Traj-2, and Traj-3, respectively; *p* < 0.001). The multivariable model revealed that the risk of death was higher in patients with Traj-3 than in patients with Traj-1 (odds ratio [OR]: 3.3, 95% confidence interval [CI]: 1.9–5.8) but similar for patients with Traj-1 and Traj-2. Similarly, the logistic analysis indicated the worst neurological outcomes in patients with Traj-3 (OR: 3.6, 95% CI: 2.0–6.4, relative to Traj-1), but similar neurological outcomes for patients in Traj-1 and Traj-2.

**Conclusion:** Persistent, but not transient severe arterial hyperoxia, was associated with poor outcome in patients with HBI.

## Introduction

Hemorrhagic brain injury (HBI), including traumatic/non-traumatic intracerebral hemorrhage, extra/subdural hemorrhage, and subarachnoid hemorrhage (SAH), has a distinct illness trajectory characterized by sudden, unexpected presentation and uncertain prognosis (1). Despite improvements in management, HBI still accounts for the death of ~10 million people every year and causes long-term disabilities (2, 3). Many risk factors for poor outcome of various type of HBI have been identified, such as glasgow coma scale (GCS) on admission, severity of illness (4), low calcium (4) and hemoglobin levels (5), high serum lactate and blood glucose levels, and a low cholinesterase level (6). Impaired oxygenation is common among patients with HBI (7, 8), and multiple studies have shown that prehospital hypoxia (9) or hypoxic episodes during intensive care (10) are associated with poor prognosis in patients with traumatic brain injury (TBI).

Given the evidence that hypoxemia is detrimental to patients with HBI, high-concentration oxygen therapy is frequently administered to patients with HBI to avoid hypoxemia, particularly to those who have just been admitted to the intensive care unit (ICU) and have not been adequately evaluated (11). However, this practice may cause hyperoxia, and there is accumulating evidence that hyperoxia on admission is associated with poorer outcomes in patients with HBI, including SAH (12–14), TBI (15, 16), and stroke (17). A study (15) of 1,547 patients with TBI found that hyperoxia (based on the average arterial oxygen partial pressure [PaO_2_]) within the first 24 h of hospitalization was associated with worse neurological outcomes and a higher mortality, and another retrospective study (17) found that hyperoxia (based on the first PaO_2_ measurement) on ICU admission was associated with higher in-hospital mortality among 2,894 ventilated patients with stroke.

However, a common limitation of these studies is that only static PaO_2_ values were evaluated. The lack of consideration of the longitudinal dynamic PaO_2_ change over time may increase the risk of bias and clinical confusion. For example, it remains unclear whether high oxygen concentration should be used in HBI patients (17).

Group-based trajectory modeling has been widely used to map symptom progression and to assess heterogeneous responses to clinical interventions (18). This study addressed the limitations of previous studies by adopting a group-based trajectory approach to analyze longitudinal PaO_2_ data of patients admitted to the ICU with various type of HBIs. We hypothesized that different PaO_2_ trajectories are associated with different clinical outcomes.

## Materials and Methods

### Data Source

Data were extracted from the Medical Information Mart for Intensive Care III (MIMIC III) database (19), an online database containing detailed information on more than 40,000 ICU patients from Beth Israel Deaconess Medical Center. Patient information has been anonymized for privacy. Author YS had access to this database (Certification number: 1564657) and was responsible for data extraction.

### Ethics Compliance

This study was performed in accordance with the ethical standards as laid down in the 1964 Declaration of Helsinki and its later amendments. Analysis of data from this database was approved by the Institutional Review Boards of the Massachusetts Institute of Technology and Beth Israel Deaconess Medical Center. Informed consent from the patients was waived due to the retrospective nature of the study.

### Study Population and Stratification Method

Preliminary screening of patients was performed by using the International Classification of Diseases, Ninth Revision (ICD 9) codes for different types of HBI, including spontaneous or traumatic intracerebral hemorrhage, SAH, and extra/subdural hemorrhage. The detailed causes of HBI could not be clearly distinguished due to the content of the ICD 9 codes. Therefore, all patients with any type of HBI were pooled in one cohort. The exclusion criteria were as follows: (i) age < 18 years, (ii) no record of PaO_2_ levels, and (iii) records of hypoxia, defined as PaO_2_ value < 60 mmHg.

### Data Extraction

Data were collected from the MIMIC III database. These data include demographic information, comorbidity, laboratory results, transfer information, disease severity, and clinical evaluation results. The first value on ICU admission was used as the initial value for laboratory indexes and hemodynamic data and the mean/maximum laboratory indexes and hemodynamic data were calculated using the mean/maximum values during the entire ICU stay. In order to conduct the trajectory modeling, PaO_2_ data were extracted from the blood gas measurements obtained within 72 h of ICU admission, with no fixed measurement time points. For patients admitted to ICU more than once, only the first admission was included.

### Construction of the Group-Based Trajectory Models

In this study, PaO_2_ levels within 72 h of ICU admission were used to identify patients with similar PaO_2_ trajectories. Mild hyperoxia was defined as patients with mild hyperoxia during the 72 h following admission to ICU; transient severe hyperoxia was defined as patients with high PaO_2_ at ICU admission that rapidly decreased to mild hyperoxia; persistent severe hyperoxia was defined as patients with persistent high PaO_2_ levels within 72 h after ICU admission. The process of constructing models was as follows: (i) the Bayesian information criterion was selected to determine the number of trajectories; (ii) the log Bayes factor [2log_e_(B10)] was used to determine whether to use a complex model or a simple model; and (iii) the average posterior probability was calculated to evaluate the posterior probability of each individual being assigned to the corresponding PaO_2_ trajectory, with an acceptable value of 0.70.

### Primary and Secondary Outcomes

In-hospital mortality was used as the primary outcome, and Glasgow Coma Scale at hospital discharge (GCS_dis_) was used as the secondary outcome. According to the GCS_dis_, patients were classified into three categories: mild (GCS_dis_: 13–15), moderate (GCS_dis_: 9–12), and severe (GCS_dis_: 3–8).

### Missing Data Management

Continuous data were missing at a frequency of <5%. Missing values were replaced by the mean or median value according the distribution of these variables. For binary data (such as gender), the missing value cannot be replaced by the mean value, and therefore was replaced by the default value.

### Statistical Analyses

Categorical variables, which are presented as percentages, were compared using the chi-square test or Fisher's exact test. Continuous variables, which are presented as means ± standard deviations (SD) or medians according to the data's distribution, were compared using Student's *t*-test or the Wilcoxon rank-sum test. Ordinary and ordered logistic regression methods were used to determine risk factors for in-hospital mortality and GCS_dis_, respectively. Multivariable logistic regression models were built as follows: first, variables for which the univariate analysis determined *p*-values of <0.20 were included (20, 21) for further multivariable analysis. Seventeen covariables were identified in this step: age, maximum Sequential Organ Failure Assessment (SOFA) score, chronic obstructive pulmonary disease (COPD), hypertension, diabetes, apnea, intubation status, white blood cell (WBC) and platelet (PLT) counts, serum calcium, potassium, sodium, blood glucose, and creatinine levels, vasopressor use, initial GCS score, and fluid balance status. Next, we used a stepwise backward elimination method to remove variables with a *p* > 0.05 (COPD, diabetes, serum potassium, and fluid balance were removed in this step). Multicollinearity was tested using the variance inflation factor (VIF ≥ 5 indicate multicollinearity) method (Serum calcium and sodium levels, and platelet count had VIFs ≥5 and hence they were removed). All statistical analyses were performed using Stata 11.2 (StataCorp, College Station, TX, USA). Two-sided *p* < 0.05 were considered to be statistically significant.

## Results

### Baseline Characteristics of Survivors and Non-survivors

A total of 2,028 patients were included in this study (overall mortality rate, 30.4%; mean age, 62 years; 56.5% male) and 724 patients were excluded due to lack of PaO_2_ data. Compared with the survivors, the non-survivors were older, and had significantly higher initial (230.3 ± 132.6 mmHg and 211.1 ± 121.2 mmHg, respectively, *p* < 0.001) and maximum (290.3 ± 123.9 mmHg and 256.4 ± 119.6 mmHg, respectively, *p* < 0.001) PaO_2_ levels. The initial and maximum GCS scores and the maximum albumin level were significantly lower, while the maximum WBC count, serum creatinine and sodium levels, fluid balance, SOFA score, and proportion with vasopressor use were significantly higher among the non-survivors. A detailed comparison of the survivors and non-survivors is shown in Table 1.

**Table 1:** Comparison of the characteristics of survivors and non-survivors.

**Demographics**	**Survivors (*n* = 1,411)**	**Non-survivors (*n* = 617)**	**Overall (*n* = 2,028)**	***p***
Age (years)	59.5 ± 19.4	68.4 ± 17.5	62.6 ± 19.3	<0.001
Male [*n* (%)]	813 (57.6)	334 (54.1)	1,147 (56.5)	0.145
Weight (kg)	78.9 ± 19.0	75.6 ± 19.7	77.9 ± 19.3	<0.001
**Comorbidities**				
Hypertension [*n* (%)]	584 (41.3)	313 (50.7)	897 (44.2)	<0.001
Diabetes mellitus [*n* (%)]	201 (14.2)	127 (20.5)	328 (16.1)	<0.001
Coronary diseases [*n* (%)]	95 (6.7)	59 (9.5)	154 (7.6)	0.027
**ICU types**				0.003
TICU [*n* (%)]	1,130 (80.0)	453 (73.4)	1,583 (78.0)	
MICU [*n* (%)]	141 (10.0)	93 (15.0)	234 (11.5)	
CSCU [*n* (%)]	104 (7.3)	48 (7.7)	152 (7.4)	
Time interval between hospital and ICU admission (days)	0.27 ± 1.8	0.29 ± 2.4	0.28 ± 2.0	0.879
Maximum WBC count (10^9^/L)	15.5 ± 6.3	17.5 ± 9.0	16.1 ± 7.3	<0.001
Minimum hemoglobin level (g/dL)	9.7 ± 1.8	9.8 ± 2.2	9.7 ± 2.0	0.134
Minimum platelet count (10^9^/L)	175.9 ± 73.3	166.9 ± 85.5	173.2 ± 77.3	0.016
Minimum albumin level (g/dL)	3.1 ± 0.5	2.9 ± 0.6	3.1 ± 0.6	<0.001
Maximum serum creatinine (mg/dL)	1.0 ± 0.9	1.4 ± 1.1	1.2 ± 1.0	<0.001
Minimum serum sodium (mmol/L)	135.3 ± 4.5	136.9 ± 6.0	135.8 ± 5.1	<0.001
Initial glucose level (mmol/L)	7.8 ± 2.2	8.6 ± 3.1	8.0 ± 2.5	<0.001
Maximum lactate level (mmol/L)	2.4 ± 1.7 (*n* = 1,054)	3.7 ± 3.3 (*n* = 469)	2.8 ± 2.4 (*n* = 1,523)	<0.001
Initial PaO_2_ level (mmHg)	211.1 ± 121.2	230.3 ± 132.6	216.9 ± 125.0	<0.001
Maximum PaO_2_ level (mmHg)	256.4 ± 119.6	290.3 ± 123.9	266.7 ± 121.9	<0.001
**Hemodynamic data**				
Fluid balance (mL/48 h/kg)	15.9 ± 46.6	32.6 ± 60.9	21.0 ± 51.9	<0.001
Vasopressor use within 48 h [*n* (%)]	97 (6.8)	141 (22.8)	238 (11.7)	<0.001
**Clinical evaluation**				
SOFA at ICU admission [median (IQR)]	3 (2-5)	5 (2-7)	3 (2-5)	<0.001
Maximum SOFA [median (IQR)]	7 (5-9)	9 (7-11)	8(5-9)	<0.001
Intubated [*n* (%)]	1,016 (72.0)	550 (89.1)	1,566 (77.2)	<0.001
Initial GCS level	8 (7-14)	6 (3-7)	7 (4-12)	<0.001

*For categorical variables, the survivors and non-survivors were compared using Chi-squared tests or Fisher's exact test. For continuous variables, the survivors and non-survivors were compared using Student's t-test or the Wilcoxon rank-sum test, according to the data distribution.*

*CSCU, cardiac surgical care unit; GCS, Glasgow Coma Scale; ICU, intensive care unit; IQR, interquartile range; LOS, length of stay; MICU, medical intensive care unit; PaO_2_, oxygen partial pressure; SOFA, Sequential Organ Failure Assessment; TICU, trauma intensive care unit; WBC, white blood cell*.

### Partial Pressure of Oxygen-Based Trajectories

**Figure 1:**
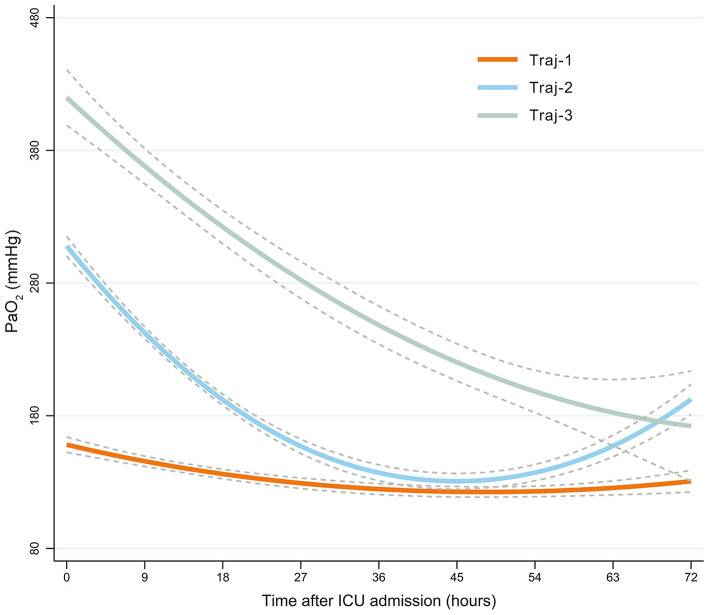
PaO_2_-based trajectories of patients with HBI. Traj-1 (mild hyperoxia), patients with mild hyperoxia during the 72 h following admission to ICU; Traj-2 (transient severe hyperoxia), patients with high PaO_2_ at ICU admission that rapidly decreased to mild hyperoxia; and Traj-3 (persistent severe hyperoxia), patients with persistent high PaO_2_ levels within 72 h after ICU admission. Group-based trajectory modeling was used to establish these PaO_2_ trajectories. PaO_2_, oxygen partial pressure; HBI, hemorrhagic brain injury; ICU, intensive care unit.

The trajectory model selection process is shown in Supplementary Table 1. A total of 12,205 PaO_2_ records were extracted, and a mean of six PaO_2_ (SD = 5.0) values were recorded for each patient. The Bayesian information criterion and statistical significance determined the selection of three trajectories (Figure 1): Traj-1 (mild hyperoxia), patients with mild hyperoxia during the 72 h following admission to ICU; Traj-2 (transient severe hyperoxia), patients with high PaO_2_ at ICU admission that rapidly decreased to mild hyperoxia; and Traj-3 (persistent severe hyperoxia), patients with persistent high PaO_2_ levels within 72 h after ICU admission. The direction of Traj-2 and Traj-3 appeared similar, and compared with Traj-2, the persistence of hyperoxia in Traj-3 was primarily attributable to the higher initial PaO_2_ level. Furthermore, according to the logarithmic Bayes factor, each of the three PaO_2_ trajectories was the product of a unique quadratic function describing PaO_2_ as a function of time. A detailed comparison of PaO_2_ characteristics is shown in Table 2.

**Table 2:** Partial pressure of oxygen indexes according to the partial pressure of oxygen trajectory.

	**Traj-1 (*n* = 1,303)**	**Traj-2 (*n* = 640)**	**Traj-3 (*n* = 85)**	***p***
PaO_2_ on admission (mmHg)	149.4 ± 68.1	332.6 ± 106.1^**^	396.7 ± 129.0^**^^,^^##^	<0.001
Maximum PaO_2_ (mmHg)	188.1 ± 75.8	367.6 ± 90.1^**^	455.8 ± 88.1^**^^,^^##^	<0.001
Minimum PaO_2_ (mmHg)	100.0 ± 39.7	141.4 ± 66.8^**^	258.3 ± 157.8^**^^,^^##^	<0.001
Mean PaO_2_ (mmHg)	132.8 ± 36.1	221.3 ± 49.8^**^	346.1 ± 110.2^**^^,^^##^	<0.001
Time to maximum PaO_2_ (h)	18.0 ± 19.6	8.2 ± 14.0^**^	17.4 ± 14.0^##^	<0.001
PaO_2_ > 150 mmHg, *n* (%)	2,220/7,610 (29.1)	2,818/4,167 (67.6)^**^	321/428 (86.7)^**^	<0.001
PaO_2_ > 200 mmHg, *n* (%)	699/7,610 (9.2)	1,543/4,167 (37.0)^**^	288/428 (67.3)^**^	<0.001
PaO_2_ > 250 mmHg, *n* (%)	271/7,610 (3.6)	924/4,167 (22.2)^**^	223/428 (52.1)^**^	<0.001
**Daily maximum PaO_2_**				
PaO_2_ on Day 1 (mmHg)	181.6 ± 73.4	363.5 ± 94.3^**^	470.1 ± 95.8^**^^,^^##^	<0.001
PaO_2_ on Day 2 (mmHg)	142.2 ± 60.0	180.3 ± 68.8^**^	334.0 ± 111.9^**^^,^^##^	<0.001
PaO_2_ on Day 3 (mmHg)	136.4 ± 48.8	176.3 ± 71.7^**^	231.7 ± 117.7^**^^,^^##^	<0.001
**Daily mean PaO_2_**				
PaO_2_ on Day 1 (mmHg)	140.1 ± 39.7	251.2 ± 53.3^**^	405.0 ± 103.6^**^^,^^##^	<0.001
PaO_2_ on Day 2 (mmHg)	122.1 ± 40.9	155.1 ± 44.6^**^	261.3 ± 81.2^**^^,^^##^	<0.001
PaO_2_ on Day 3 (mmHg)	117.5 ± 32.1	149.4 ± 44.4^**^	187.0 ± 62.7^**^^,^^##^	<0.001
Number of PaO_2_ measurements	5.8 ± 5.2	6.5 ± 4.5^*^	5.0 ± 4.3^#^	0.004

*One-way analysis of variance was used to compare the trajectories. The p-values represent the difference in trend between groups. The Bonferroni correction was used to adjust the p-values for multiple comparisons between two groups.*

* *p < 0.05 compared with Traj-1;*

** *p < 0.01 compared with Traj-1;*

# *p < 0.05 compared with Traj-2;*

## *p < 0.01 compared with Traj-2.*

*PaO_2_, partial pressure of oxygen, Traj-1, mild hyperoxia; Traj-2, transient severe hyperoxia; Traj-3, persistent severe hyperoxia*.

### Mechanical Parameters According to the Partial Pressure of Oxygen Trajectory

The mechanical parameters according to PaO_2_ trajectory, including fraction of inspired oxygen (FiO_2_), minute ventilation, tidal volume, positive end expiratory pressure (PEEP), and respiratory rate, are shown in Table 3. The initial FiO_2_ (within 3 h after ICU admission) was comparable between Traj-2 and Traj-3, and was significantly higher in the Traj-2 and Traj-3 groups than that in the Traj-1 group (79 ± 24, 81 ± 26, and 65 ± 24 for Traj-2, Traj-3, and Traj-1 groups, respectively, *p* <0.001). However, both the mean and maximum FiO_2_ were significantly higher in the Traj-3 group than those in the Traj-1 and Traj-2 groups. Compared with Traj-1, the initial/mean minute ventilation, the maximum tidal volume, the initial/mean/maximum PEEP, and the initial and mean respiratory rate were significantly lower in Traj-2, and the initial/mean/maximum minute ventilation, maximum tidal volume, initial/mean/maximum PEEP, and the initial and mean respiratory rate were significantly lower in Traj-3. There were no significant differences between Traj-2 and Traj-3 in the minute ventilation, tidal volume, PEEP, or the respiratory rate.

**Table 3:** Respiratory parameters according to the partial pressure of oxygen trajectory.

	**Traj-1**	**Traj-2**	**Traj-3**	***p***
**FiO_2_(%)**				
Initial FiO_2_	65.9 ± 24.3 (*n* = 481)	79.5 ± 24.5 (*n* = 282)^**^	81.8 ± 26.2 (*n* = 33)^**^	<0.001
Mean FiO_2_	54.0 ± 15.2	53.4 ± 15.1	66.0 ± 21.2^**^^,^^##^	<0.001
Maximum FiO_2_	71.6 ± 25.2	78.3 ± 25.3^**^	91.1 ± 19.4^**^^,^^##^	<0.001
**MV (L/min)**				
Initial MV	9.6 ± 2.9 (*n* = 988)	9.2 ± 8.3 (*n* = 603)^*^	8.4 ± 2.5 (*n* = 76)^**^	<0.001
Mean MV	9.6 ± 2.3	8.9 ± 2.3^**^	8.5 ± 1.8^**^	<0.001
Maximum MV	12.8 ± 3.6	12.2 ± 8.4	11.2 ± 3.3*	0.021
**V_t_ (ml)**				
Initial V_t_	545 ± 123 (*n* = 966)	541 ± 113 (*n* = 599)	517 ± 125 (*n* = 74)	0.144
Mean V_t_	547 ± 96	540 ± 98	533 ± 94	0.255
Maximum V_t_	647 ± 126	630 ± 125*	609 ± 126*	0.003
**PEEP (cmH_2_O)**				
Initial PEEP	6.5 ± 2.9 (*n* =275)	5.5 ± 1.5 (*n* = 174)^*^	5.2 ± 0.3 (*n* = 26)^*^	<0.001
Mean PEEP	6.4 ± 2.6	5.6 ± 1.4^**^	5.3 ± 0.4^*^	<0.001
Maximum PEEP	7.1 ± 3.3	6.2 ± 2.1^**^	5.5 ± 1.0^*^	<0.001
**RR (times/min)**				
Initial RR	18.9 ± 5.1 (*n* = 1,206)	18.1 ± 5.1 (*n* = 622)^*^	17.3 ± 5.7 (*n* = 81)^**^	<0.001
Mean RR	19.1 ± 6.6	17.7 ± 3.2^**^	16.5 ± 3.4^**^	<0.001
Maximum RR	29.2 ± 3.5	26.3 ± 9.5	24.1 ± 7.0	0.083

*One-way analysis of variance was used to compare the trajectories. The p-values represent the difference in the trend between groups. The Bonferroni correction was used to adjust the p-values for multiple comparisons between two groups.*

* *p < 0.05 compared with Traj-1.*

** *p < 0.01 compared with Traj-1.*

*#p < 0.05 compared with Traj-2.*

## *p < 0.01 compared with Traj-2.*

*FiO_2_, fraction of inspired oxygen; MV, minute ventilation; PEEP, positive end expiratory pressure; RR, respiratory rate; Traj-1, mild hyperoxia; Traj-2, transient severe hyperoxia; Traj-3, persistent severe hyperoxia; Vt, tidal volume*.

### Clinical Outcomes According to the Partial Pressure of Oxygen Trajectory

**Figure 2:**
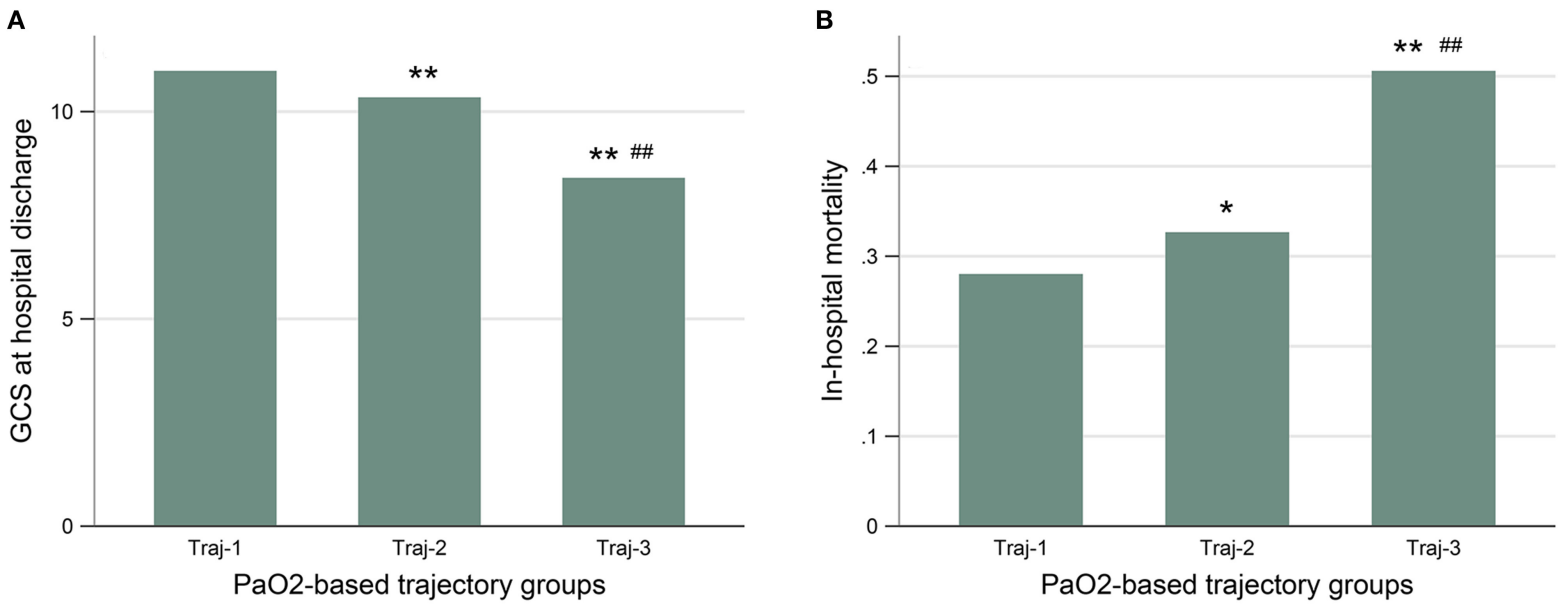
The GCS at hospital discharge **(A)**, and the in-hospital mortality **(B)** were similar between patients with Traj-1 and Traj-2, both of which were significantly lower than that in patients with Traj-3. GCS, Glasgow Coma Scale; Traj-1, mild hyperoxia; Traj-2, transient severe hyperoxia; Traj-3, persistent severe hyperoxia. Kruskal–Wallis test was used for GCS to compare the three trajectories and the nemenyi test was used to adjust the *p*-values for multiple comparisons between two groups. The chi-square test was used for in-hospital mortality. The *p*-values represent the difference in trend between groups. *p < 0.05 compared with Traj-1. **p < 0.01 compared with Traj-1. ^#^*p* < 0.05 compared with Traj-2. ^##^*p* < 0.01 compared with Traj-2.

The patients with Traj-1 had the lowest in-hospital mortality and best neurological outcomes (Figure 2 and Supplementary Table 2). Although the initial and the maximum PaO_2_ level were similar in Traj-2 and Traj-3, the mortality was significantly higher, and the GCS_dis_ level was significantly lower in Traj-3 (in-hospital mortality: 32.7 and 50.6%; GCS_dis_: 11 and 7 for Traj-2 and Traj-3, respectively; both *p* < 0.001). Detailed comparisons of the three trajectories are provided in Supplementary Table 2. The result of univariate analysis is provided in Supplementary Table 3. In the multivariable logistic regression model (Table 4), relative to Traj-1 group, the risk of mortality was significantly higher in the Traj-3 group (odds ratio [OR]: 3.3, 95% confidence interval [CI]: 1.9–5.8) but not in the Traj-2 group (OR: 1.2, 95% CI: 0.9–1.5). Similarly, the logistic regression analysis (Table 4) revealed that, relative to the Traj-1 group, the neurological outcomes were significantly worse in the Traj-3 group (OR: 3.6, 95% CI: 2.0–6.4) but not in the Traj-2 group (OR: 1.0, 95% CI: 0.8–1.3). In addition, age > 65 years, intubation, hypertension, diabetes, SOFA score, high initial blood glucose, and maximum WBC count were significant risk factors for in-hospital mortality and a poor neurological outcome, while apnea and high GCS on admission were associated with lower in-hospital mortality and a better neurological outcome.

**Table 4:** Associations between the partial pressure of oxygen trajectory and prognosis in multivariable logistic regression models.

**In-hospital mortality (Model A)**			**Poor neurological outcome (Model B)**		
**Variables**	**Adjusted OR (95% CI)**	***P***	**Variables**	**Adjusted OR (95% CI)**	***p***
Traj-1	Reference	–	Traj-1	Reference	–
Traj-2	1.2 (0.9–1.5)	0.101	Traj-2	1.0 (0.8–1.3)	0.532
Traj-3	3.3 (1.9–5.8)	<0.001	Traj-3	3.6 (2.0–6.4)	<0.001
Age > 65 (years)	3.2 (2.5–4.1)	<0.001	Age > 65 (years)	2.7 (2.1–3.4)	<0.001
Intubated	1.4 (1.1–1.8)	0.006	Intubated	1.5 (1.1–1.9)	0.001
Hypertension	1.5 (1.2–1.9)	0.001	Hypertension	1.3 (1.1–1.7)	0.009
Initial glucose level	1.006 (1.003–1.008)	<0.001	Initial glucose level	1.008 (1.040–1.009)	<0.001
Apnea	0.2 (0.1–0.5)	0.001	Apnea	0.3 (0.1–0.7)	0.009
Maximum SOFA score	1.2 (1.1–1.2)	<0.001	Maximum SOFA score	1.2 (1.1–1.3)	<0.001
Maximum WBC count	1.02 (1.00–1.03)	0.008	Maximum WBC count	1.02 (1.00–1.03)	0.010
Maximum creatinine level	1.2 (1.1–1.3)	0.001	Maximum creatinine level	1.1 (1.0–1.2)	0.021
GCS on admission	0.8 (0.8–0.8)	<0.001	GCS on admission	0.8 (0.7–0.8)	<0.001

*Model A used in-hospital mortality as the dependent variable and Model B used poor neurological outcome as the dependent variable. The variance inflation factor was 3.4 and 3.3 for Models A and B, respectively.*

*CI, confidence interval; GCS, Glasgow Coma Scale; OR, odds ratio; SOFA, Sequential Organ Failure Assessment; Traj-1, mild hyperoxia; Traj-2, transient severe hyperoxia; Traj-3, persistent severe hyperoxia; WBC, white blood cell*.

## Discussion

The current study has two major findings. First, based on the longitudinal PaO_2_ data, we identified three PaO_2_ trajectories in patients with HBI, with significantly different clinical outcomes. Second, persistent hyperoxia was associated with poor outcomes in patients with HBI, while transient hyperoxia was not.

Impaired pulmonary oxygenation is common among patients with HBI (9, 22, 23). However, as there is evidence that cerebral ischemia can be alleviated by increasing PaO_2_ levels to ensure adequate oxygen delivery to the brain (12), high-concentration oxygen therapy is commonly applied to brain-injured patients, especially in those who have not been adequately evaluated (11), to prevent hypoxia (24, 25). One recent guideline (11) on oxygen therapy suggests that initial oxygen therapy with 15 L/min with a reservoir mask should be provided to patients before oximetry data are obtained. However, these strategies often lead to transient hyperoxia, and the role of hyperoxia in the neurocritical care setting remains controversial. Most previous studies (13–16, 26) have found that hyperoxia was associated with poorer neurological outcomes and increased mortality. For example, in a retrospective analysis of 3,420 patients with TBI, Davis et al. (16) found that both hypoxia and hyperoxia on hospital arrival in severe TBI were associated with increased mortality and a decrease in neurological function, compared with normoxia. Another retrospective study (26) in TBI also found that an elevated first PaO_2_ value during the first 24 h in ICU was associated with higher in-hospital mortality. In addition, Rincon et al. (17) reported that in patients with ventilated stroke (both ischemic and hemorrhagic), PaO_2_ ≥ 300 mmHg was independently associated with an increased risk of in-hospital death compared with patients with either normoxia or hypoxia. Similar findings have been reported in patients with SAH (13, 14). However, other studies with different findings were also reported. In a national multicenter cohort study (27) of 3,033 adult patients with spontaneous ICH, Fallenius et al. (27) found no significant relation between the PaO_2_ levels (hyperoxia: > 150 mmHg) and long-term mortality. Another study (28) found that early moderate hyperoxia (>150 mmHg) did not increase or decrease the risk of a poor outcome in mechanically ventilated patients with SAH.

Different definitions for hyperoxia may be one important reason for these inconsistent findings. Previous studies that investigated the effect of hyperoxia on patients with different types of HBI used different definitions of hypoxia (PaO_2_ ≥ 150, >200, >300, and >487 mmHg), based on single PaO_2_ values measured at different time points, such as the first PaO_2_ within the first 24 h after admission, PaO_2_ value from the Acute Physiology and Chronic Health Enquiry score on the first day, and mean PaO_2_ (Supplementary Table 4). In this study, we explored the PaO_2_ trajectories in real clinical situations. We noted that the overall PaO_2_ was high in the whole cohort of HBI. In clinical practice, physicians may be mostly concerned about avoiding hypoxia and give additional oxygen as a precaution, as the deleterious effects of hypoxia are well-known (29, 30). Therefore, supplementary oxygen is routinely applied to brain-injured patients, even in those with adequate oxygen saturation, which may lead to transient or persistent hyperoxia.

In addition, another limitation of previous studies on the associations between hyperoxia and HBI outcome is that hyperoxia was mainly defined using a single PaO_2_ value, which may not have reflected the patients' daily PaO_2_ status. In studies that based the definition of hyperoxia on a single measurement value, both the transient and persistent hyperoxia were classified as hyperoxia. To solve this limitation, several strategies have been proposed to reflect the overall PaO_2_ status, such as average PaO_2_ (15), TWA-PaO_2_ (13, 28), and oxygen burden (14). For example, in a single-center retrospective study with 197 patients with SAH, Fukuda et al. (13) reported that a high TWA-PaO_2_ during the first 24 h was associated with delayed cerebral ischemia and a poor prognosis. Another study (14) of 252 patients with SAH also found that a high oxygen burden (≥173 mmHg) was significantly associated with delayed cerebral ischemia. Although these analytic approaches consider the overall oxygenation status, they still have some notable limitations, such as: (i) hyperoxia and hypoxia status may have been normalized as a result of being averaged (15); (ii) only static PaO_2_ values were used in the analysis; and (iii) complex calculations (TWA-PaO_2_, oxygen burden) were used, and yet, these studies did not assess the relative effects of prolonged and transient hyperoxia in patients with HBI.

In the current study, three PaO_2_ trajectories were identified based on longitudinal PaO_2_ data, using a group-based trajectory modeling approach (18). The HBI patients with mild hyperoxia had the highest survival rate and the most favorable neurological outcomes. This finding is consistent with previous reports showing that a moderately high PaO_2_ is associated with favorable outcomes in patients with TBI (16, 26). There were some notable differences between the Traj-2 and Traj-3 groups. Although the PaO_2_ on ICU admission and the maximum value were similar in the Traj-2 and Traj-3 groups, the outcome of the two trajectories differed considerably. Compared with the Traj-2 group, the Traj-3 group had a more gradual decline in the PaO_2_ level, and was associated with a lower GCS score and a lower survival rate. This suggests that in patients with HBI, transient hyperoxia is not detrimental and that only persistent hyperoxia is associated with poor outcomes.

The reason behind the different PaO_2_ status within these trajectories remains unclear. We noted that the initial FiO_2_ was comparable between the Traj-2 and Traj-3 groups, and was significantly higher than that in the Traj-1 group. This is consistent with above finding that the initial PaO_2_ was significantly higher in the Traj-2 and Traj-3 groups than that in the Traj-1 group. In addition, both the mean and maximum FiO_2_ were significantly higher in the Traj-3 group than those in Traj-1 and Traj-2 groups. This also corresponds to the subsequent PaO_2_ changes within these trajectories. In addition, we found that other mechanical parameters including minute ventilation, tidal volume, and PEEP were comparable within the three trajectories; therefore, hyperoxia was mainly caused by high FiO2 levels. Our data suggest that persistent hyperoxia, which is associated with a poor outcome, may be avoided by proper adjustment of the FiO_2_ levels.

The mechanism between hyperoxia and poor prognosis cannot be inferred from this study. Under experimental conditions (31), hyperoxia can decrease heart rate and cardiac output. Studies in healthy subjects have found that hyperoxia is associated with decreased cerebral blood flow (32). Therefore, in addition to increased radical oxygen species (33), hyperoxia could cause decreased oxygen delivery to the brain-injured region if the increase in arterial O_2_ level during hyperoxia is counterbalanced by a decrease in cardiac output and cerebral flow. More studies are needed to explore the potential mechanism.

We identified several factors associated with prognosis in the multivariable logistic regression analysis, including age > 65 years, intubation, hypertension, diabetes, apnea, SOFA score, glucose level, WBC count, and GCS on admission. These results are in line with previous findings that age (34), intubation (35), GCS on admission, severity of illness (4), hypertension, diabetes (36), apnea, high blood glucose levels (6), and WBC count (37) are significant predictors of the outcomes in patients with various HBI.

### Strengths and Limitations

To the best of our knowledge, this study is the first to evaluate the association between the dynamic PaO_2_ changes and the clinical outcomes in patients with HBI using a PaO_2_-based trajectory model. The study has some limitations. First, only short-term outcomes were evaluated, and the relationship between transient/prolonged exposure to PaO_2_ and long-term outcomes, such as the 3-month GOS and the modified Rankin score, were not investigated due to a lack of data. Second, the old ICD 9 codes were used to identify patients with HBI in the current study. However, the bleeding type of these patients cannot be inferred due to the content of the ICD 9 codes. Therefore, patients with different types of HBI were pooled in one cohort. Third, although we considered the data on all the available confounders, there may be some residual confounding because some unmeasured potential confounders (e.g., surgery details and intracranial pressure) were not included in the multivariable logistic regression models. In addition, other factors such as GCS, age, intubation status also had significant associations with the prognosis. These findings should be interpreted with caution. Fourth, the data were collected from 2001 to 2012, which hinders the interpretation of these results as the findings may have changed over time due to changes in clinical management. However, as hyperoxia is a clinical phenomenon rather than a clinical treatment strategy, changes in treatment strategies since 2012 are not likely to have altered the effect of hyperoxia on the prognosis of patients with HBIs. Fifth, the time period between onset of the HBI and hospital admission may have a significant association with the prognosis of these patients. However, this information was not available in the study database. In addition, PaCO_2_ is an important index reflecting respiratory status and may influence intracranial physiology. However, due to the lack of data, the potential confounding effects of PaCO_2_ were not investigated. Finally, the number of patients in the Traj-3 group was small, and a validation cohort was consequently not isolated from the whole cohort.

## Conclusions

We identified three PaO_2_ trajectories among patients with HBI who were admitted to the ICU. Patients with transient hyperoxia and normoxia had similar clinical outcomes, while those with persistent hyperoxia had worse neurological outcomes and higher mortality rates than did the patients from the other two groups. Further studies are needed to validate our findings and to determine whether there is a causal relationship between the PaO_2_ trajectory and the outcome in patients with HBI. Also, the association between the PaO_2_ trajectory and intracranial physiology and long-term outcomes needs to be further investigated.

## Data Availability Statement

The raw data supporting the conclusions of this article will be made available by the authors, without undue reservation.

## Ethics Statement

The studies involving human participants were reviewed and approved by Institutional Review Boards of the Massachusetts Institute of Technology and Beth Israel Deaconess Medical Center, USA. Written informed consent for participation was not required for this study in accordance with the national legislation and the institutional requirements.

## Author Contributions

GC and JY designed the study. QX and JW analyzed the data and prepared the figures. YS and SG collected and verified all data. WR and YS wrote the draft of the manuscript. JW made critical revisions. All authors gave final approval of the version to be published and agreed to be accountable for all aspects of the work in ensuring that questions related to the accuracy or integrity of any part of the work are answered.

## Funding

This work was supported by a grant from the Zhejiang Medical Association (No. 2019ZYC-A73) to YS. GC also received a funding from Zhejiang Medicine and Health Science and Technology Project (No. WKJ-ZJ-2001).

## Conflict of Interest

The authors declare that the research was conducted in the absence of any commercial or financial relationships that could be construed as a potential conflict of interest.

## Publisher's Note

All claims expressed in this article are solely those of the authors and do not necessarily represent those of their affiliated organizations, or those of the publisher, the editors and the reviewers. Any product that may be evaluated in this article, or claim that may be made by its manufacturer, is not guaranteed or endorsed by the publisher.
