# Supplementary material for: Association Between Oxygen Partial Pressure Trajectories and Short-Term Outcomes in Patients With Hemorrhagic Brain Injury
Source: Front Med (Lausanne). 2021 Sep 9;8:681200. doi: 10.3389/fmed.2021.681200 (PMC8458649; doi:10.3389/fmed.2021.681200)
Supplement: Supplementary file 1 [file Table_1.DOCX]

Supplementary Material

# Supplementary Tables

**Supplementary Table S1** Selection of PaO_2_-based trajectory models

| Group-based trajectory model | BIC | 2log_e_(B_10_) | AvePP | | | |
| --- | --- | --- | --- | --- | --- | --- |
|  |  |  | Traj-1 | Traj-2 | Traj-3 | Traj-4 |
| One trajectory group | -71100 | 14.3 | 1.0 ± 0.0 (n = 2028) |  |  |  |
| Two trajectory groups | -70056 | 11.2 | 0.90 ± 0.13 (n = 1357) | 0.89 ± 0.14 (n = 671) |  |  |
| Three trajectory groups | -69784 | Reference. | 0.87 ± 0.14 (n = 1303) | 0.86 ± 0.15 (n = 640) | 0.83 ± 0.18 (n = 85) |  |
| Four trajectory groups | NS | NS | - | - | - | - |

Abbreviations: PaO2, oxygen partial pressure; BIC, Bayesian information criterion; AvePP, average posterior probability; NS, not significant.

**Supplementary Table S2** Comparison of the characteristics between three oxygen partial pressure trajectories

| Demographics | Traj-1 (n = 1303) | Traj-2 (n = 640) | Traj-3 (n = 85) | p |
| --- | --- | --- | --- | --- |
| Age (years) | 63.6 ± 18.2 | 59.3 ± 20.7 | 61.4 ± 22.0 | <0.001 |
| Male [n (%)] | 747 (57.3) | 355 (55.4) | 45 (52.9) | 0.584 |
| Hypertension [n (%)] | 610 (46.8) | 246 (38.4) | 41 (48.2) | 0.002 |
| Diabetes mellitus [n (%)] | 236 (18.1) | 81 (12.6) | 11 (12.9) | 0.006 |
| Laboratory indexes |  |  |  |  |
| Maximum white blood cell count (10^9/L) | 16.1 ± 7.6 | 16.2 ± 6.7 | 16.3 ± 6.3 | 0.885 |
| Minimum hemoglobin level (g/dL) | 9.9 ± 1.9 | 9.4 ± 2.0 | 10.0 ± 2.3 | <0.001 |
| Minimum platelet count (10^9/L) | 173.0 ± 77.3 | 172.8 ± 77.9 | 178.0 ± 73.9 | 0.836 |
| Maximum serum creatinine (mg/dL) | 1.2 ± 1.0 | 1.1 ± 0.9 | 1.1 ± 0.5 | 0.181 |
| Minimum serum sodium (mmol/L) | 144.5 ± 5.7 | 145.4 ± 6.6 | 146.4 ± 7.5 | <0.001 |
| Hemodynamic data |  |  |  |  |
| Fluid balance (mL/48 h/kg) | 23.2 ± 50.5 | 17.4 ± 55.2 | 12.7 ± 44.8 | 0.020 |
| Vasopressor-use within 48 h [n (%)] | 154 (11.8) | 75 (11.7) | 9 (10.5) | 0.943 |
| Clinical evaluation |  |  |  |  |
| SOFA at ICU admission [median (IQR)] | 4 (2–6) | 3 (1–5) | 2 (1–4) | <0.001 |
| Initial GCS level | 8 (6–14) | 6 (3–9) | 6 (4–13) | <0.001 |
| GCS at hospital discharge | 13 (7–15) | 11 (6–15) | 7 (3–14) | <0.001 |
| 3–8 | 370 (28.3) | 211 (32.9) | 45 (52.9) |  |
| 9–12 | 236 (18.1) | 129 (20.1) | 13 (15.3) |  |
| 13–15 | 697 (53.4) | 300 (46.8) | 27 (31.7) |  |
| Hospital mortality [n (%)] | 365 (28.0) | 209 (32.6) | 43 (50.6) | <0.001 |

Traj-1 (mild hyperoxia), patients with mildly elevated PaO_2_ during the 72 hours following admission to ICU; Traj-2 (transient severe hyperoxia), patients with high PaO_2_ at ICU admission that rapidly descended to normal levels; and Traj-3 (persistent severe hyperoxia), patients with high PaO_2_ levels at ICU admission that slowly descended to normal levels.

Abbreviations: PaO2, oxygen partial pressure; ICH, intracranial hemorrhage; SOFA, sequential organ failure assessment; ICU, intensive care unit; IQR, interquartile range; GCS, Glasgow Coma Scale.

**Supplementary Table S3** unadjusted associations between risk factors and prognosis in univariate logistic regression

| Model A | | | Model B | | |
| --- | --- | --- | --- | --- | --- |
| Variables | unadjusted OR (95% CI) | p | Variables | unadjusted OR (95% CI) | p |
| Traj-1 | Reference. | - | Traj-1 | Reference. | - |
| Traj-2 | 1.2 (1.0–1.5) | 0.035 | Traj-2 | 1.2 (1.0–1.5) | 0.039 |
| Traj-3 | 2.6 (1.6–4.1) | <0.001 | Traj-3 | 2.8 (1.8–4.4) | <0.001 |
| Age > 65 (years) | 2.4 (2.0–2.9) | <0.001 | Age > 65 (years) | 1.9 (1.6–2.3) | <0.001 |
| Intubated | 2.6 (2.2–3.2) | <0.001 | Intubated | 3.2 (2.7–4.0) | 0.001 |
| Hypertension | 1.4 (1.2–1.7) | <0.001 | Hypertension | 1.2 (1.0–1.5) | 0.009 |
| Diabetes | 1.5 (1.2–1.9) | <0.001 | Diabetes | 1.3 (1.0–1.7) | 0.010 |
| Apnea | 0.3 (0.1–0.6) | 0.002 | Apnea | 0.4 (0.2–0.8) | 0.015 |
| Maximum SOFA score | 1.2 (1.2–1.3) | <0.001 | Maximum SOFA score | 1.2 (1.1–1.2) | <0.001 |
| Maximum WBC count | 1.03 (1.02–1.04) | <0.001 | Maximum WBC count | 1.03 (1.02–1.05) | <0.001 |
| Maximum creatinine level | 1.3 (1.2–1.5) | <0.001 | Maximum creatinine level | 1.2 (1.1–1.4) | <0.001 |
| GCS on admission | 0.8 (0.8–0.8) | <0.001 | GCS on admission | 0.7 (0.7–0.8) | <0.001 |

Note: model A used in-hospital mortality as the dependent outcome and model B used poor neurological outcome as the dependent outcome.

Abbreviations: OR, odds ratio; CI, confidence interval; SOFA, sequential organ failure assessment; WBC, white blood cell; GCS, Glasgow Coma Scale; VIF, variance inflation factor.

**Supplementary Table S4** PaO_2_ extraction details in studies investigating the impact of hyperoxia in patients with cerebral disorders

| **Author-year** | **Location** | **PaO_2_ used in the study** | **Definition of hyperoxia** | **Definition of normoxia** | **Cohort** |
| --- | --- | --- | --- | --- | --- |
| Davis-2009 (1) | USA | First 24 h, first PaO_2_ | PaO_2_ >487 mmHg (n = 1000) | PaO_2_ 110–487 mmHg (n = 2081) | TBI |
| Brenner-2012 (2) | USA | First 24 h, mean PaO_2_ | PaO_2_ >200 mmHg (n = 666) | PaO_2_ 100–200 mmHg (n = 778) | TBI |
| Asher-2013 (3) | USA | First 72 h, highest PaO_2_ | PaO_2_ ≥200 mmHg (n = 132) | PaO_2_ 60–200 mmHg (n = 14) | TBI |
| Raj-2013 (4) | Finland | First 24 h, PaO_2_ from APACHE score | PaO_2_ >100 mmHg (n = 567) | PaO_2_ 75–100 mmHg (n = 375) | TBI |
| Rincon-2014 (5) | USA | First 24 h, first PaO_2_ | PaO_2_ ≥300 mmHg (n = 256) | PaO_2_ 60–300 mmHg (n = 403) | TBI |
| Lang-2016 (6) | Finland | First 24 h, TWA-PaO_2_ | PaO_2_ ≥150 mmHg (n = 104) | PaO_2_ 97.5–150 mmHg (n = 192) | SH |
| Fukuda-2019 (7) | Japan | First 24 h, TWA-PaO_2_ | Multiple groups | | SH |
| Jeon-2014 (8) | USA | Oxygen burden (area under the curve of PaO_2_) | Multiple groups | | SH |
| Young-2012 (9) | Australia | First 24 h, PaO_2_ from APACHE score | Multiple groups | | Stroke |
| Rincon-2014 (10) | USA | First 24 h, first PaO_2_ | PaO_2_ ≥300 mmHg (n = 450) | PaO_2_ 60–300 mmHg (n = 1316) | Stroke |
| Fallenius-2016 (11) | Finland | First 24 h, PaO_2_ from APACHE score | PaO_2_ >150 mmHg (n = 218) | PaO_2_ <97.5 mmHg (n = 892) | Stroke |

Abbreviations: PaO2, oxygen partial pressure; TBI, traumatic brain injury; SH, subarachnoid hemorrhage; APACHE, Acute Physiology and Chronic Health Enquiry; TWA-PaO2, time-weighted average PaO2.

Note: We reviewed the results of previous studies that investigated the impact of hyperoxia on patients with traumatic brain injury, SAH, or stroke. A total of 11 studies were identified. A variety of definitions of hyperoxia were used, based on PaO2 measured at variable time-points: Three studies used the first PaO2 within the first 24 h after admission, three used the PaO2 value from the Acute Physiology and Chronic Health Enquiry score on the first day, and four studies used other definitions of hyperoxia, including the mean PaO2, the time-weighted average PaO2 (TWA-PaO2), and the oxygen burden (area under the curve of PaO2).

References

1. Davis DP, Meade W, Sise MJ, et al. Both hypoxemia and extreme hyperoxemia may be detrimental in patients with severe traumatic brain injury. *J Neurotrauma*. 2009;26(12):2217-2223.

2. Brenner M, Stein D, Hu P, Kufera J, Wooford M, Scalea T. Association between early hyperoxia and worse outcomes after traumatic brain injury. *Arch Surg.* 2012;147(11):1042-1046.

3. Asher SR, Curry P, Sharma D, et al. Survival advantage and PaO2 threshold in severe traumatic brain injury. *J Neurosurg Anesthesiol*. 2013;25(2):168-173.

4. Raj R, Bendel S, Reinikainen M, et al. Hyperoxemia and long-term outcome after traumatic brain injury. *Crit Care* (London, England). 2013;17(4):R177.

5. Rincon F, Kang J, Vibbert M, Urtecho J, Athar MK, Jallo J. Significance of arterial hyperoxia and relationship with case fatality in traumatic brain injury: a multicenter cohort study. *J Neurol Neurosurg Psychiatry*. 2014;85(7):799-805.

6. Lang M, Raj R, Skrifvars MB, et al. Early Moderate Hyperoxemia does not predict outcome after aneurysmal subarachnoid hemorrhage. *Neurosurgery*. 2016;78(4):540-545.

7. Fukuda S, Koga Y, Fujita M, et al. Hyperoxemia during the hyperacute phase of aneurysmal subarachnoid hemorrhage is associated with delayed cerebral ischemia and poor outcome: a retrospective observational study. *J Neurosurg*. 2019:1-8.

8. Jeon SB, Choi HA, Badjatia N, et al. Hyperoxia may be related to delayed cerebral ischemia and poor outcome after subarachnoid hemorrhage. *J Neurol Neurosurg Psychiatry*. 2014;85(12):1301-1307.

9. Young P, Beasley R, Bailey M, et al. The association between early arterial oxygenation and mortality in ventilated patients with acute ischemic stroke. *Crit Care Resusc*. 2012;14(1):14-19.

10. Rincon F, Kang J, Maltenfort M, et al. Association between hyperoxia and mortality after stroke: a multicenter cohort study. *Crit Car Med.* 2014;42(2):387-396.

11. Fallenius M, Raj R, Reinikainen M, Bendel S, Skrifvars MB. Association between high arterial oxygen tension and long-term survival after spontaneous intracerebral hemorrhage. *Crit Car Med.* 2016;44(1):180-187.
